# Supplementary material for: Slug regulates the Dll4-Notch-VEGFR2 axis to control endothelial cell activation and angiogenesis
Source: Nat Commun. 2020 Oct 26;11:5400. doi: 10.1038/s41467-020-18633-z (PMC7588439; doi:10.1038/s41467-020-18633-z)
Supplement: Supplementary file 1 — Supplementary Information [file 41467_2020_18633_MOESM1_ESM.pdf]

Supplementary Information

Slug Regulates the Dll4-Notch-VEGFR2 Axis to Control Endothelial Cell Activation and Angiogenesis – Hultgren et al.

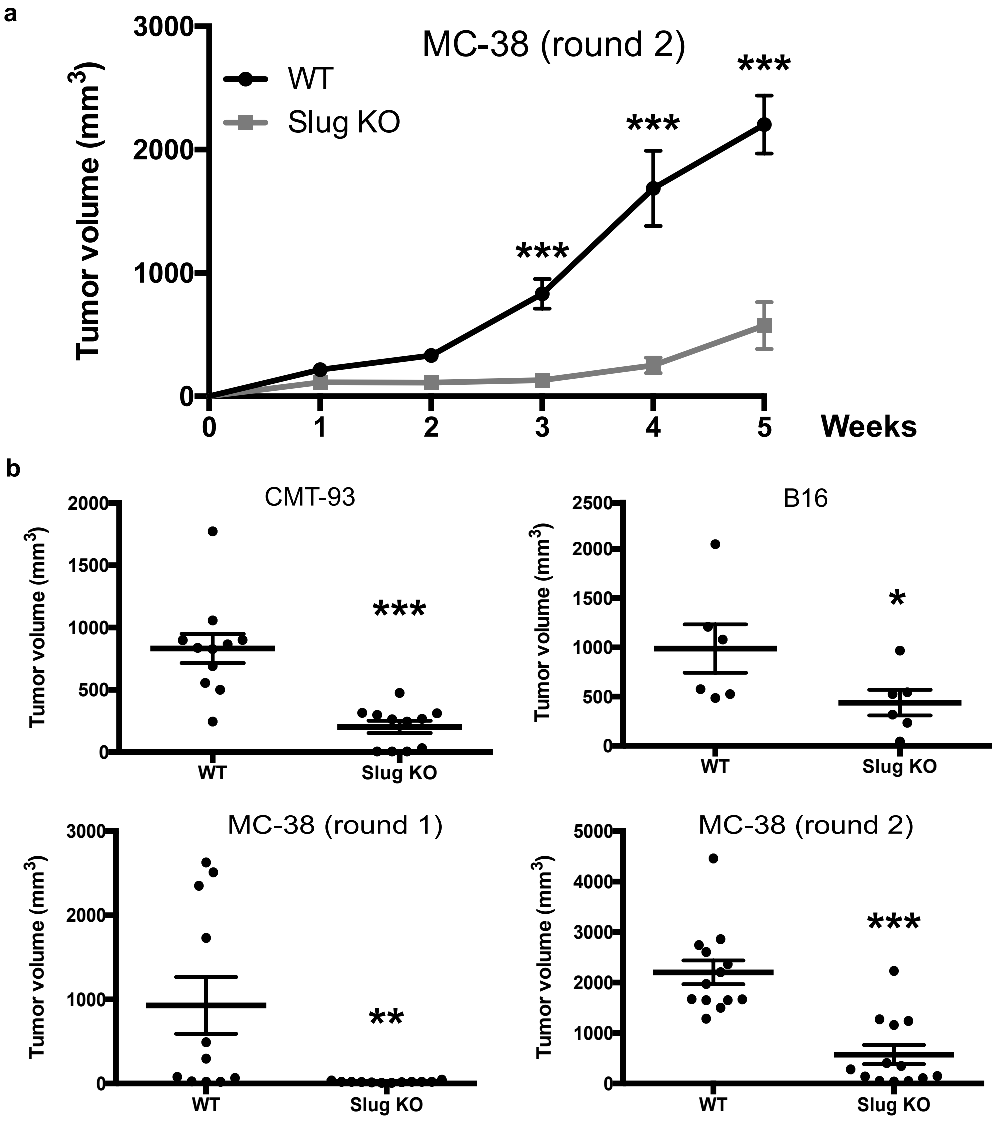

Supplementary Figure 1: Tumor growth in WT vs. SlugKO mice. a) Growth curve of MC-38 colon tumors in WT (n=13) and SlugKO (n=13) mice, experiment round 2. b) Endpoint CMT-93 (n=11 for both conditions), MC-38 Round 1 (WT: n=11, SlugKO: n=12), MC-38 Round 2 (n=13 for both conditions) and B16 (n=6 for both conditions) tumor size in WT and SlugKO mouse. As tumors grew at a different rate, endpoint was at the IACUC-mandated maximum size. Data represent mean ± SEM. Two-tailed unpaired equal-variance t-test for all except for B16 (one-tailed unpaired equal-variance t-test). *p<0.05, **p<0.01, ***p<0.005. Source data are provided as a Source Data file.

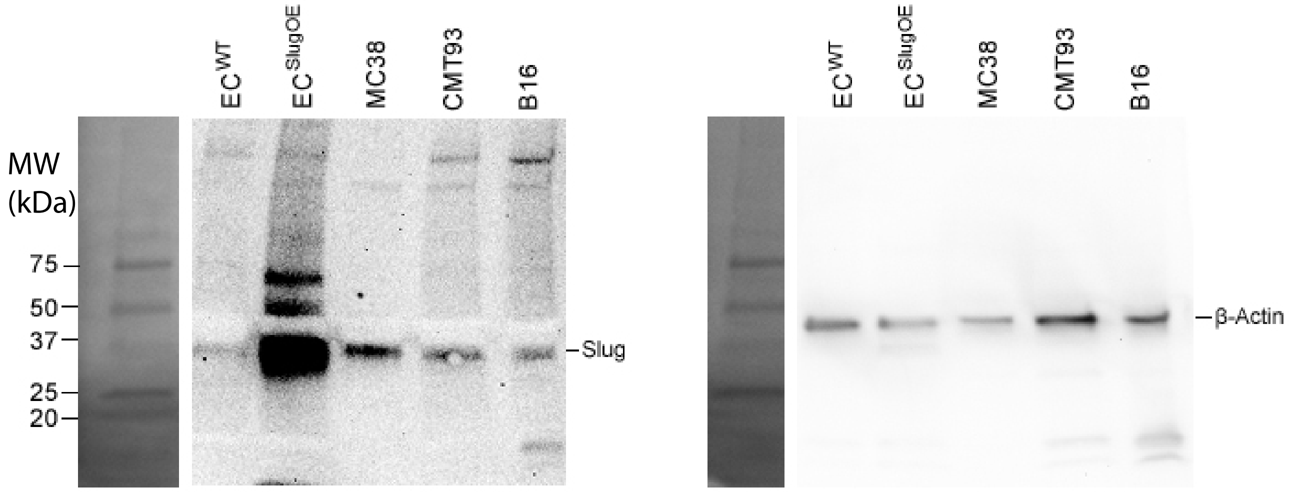

Supplemental Figure 2: Slug protein expression in tumor cell lines. Full western blots for Slug and beta-actin expression in control and SlugOE EC as well as wild type MC-38, CMT-93 and B16 tumors in vitro. This experiment was conducted once Source data are provided as a Source Data file.

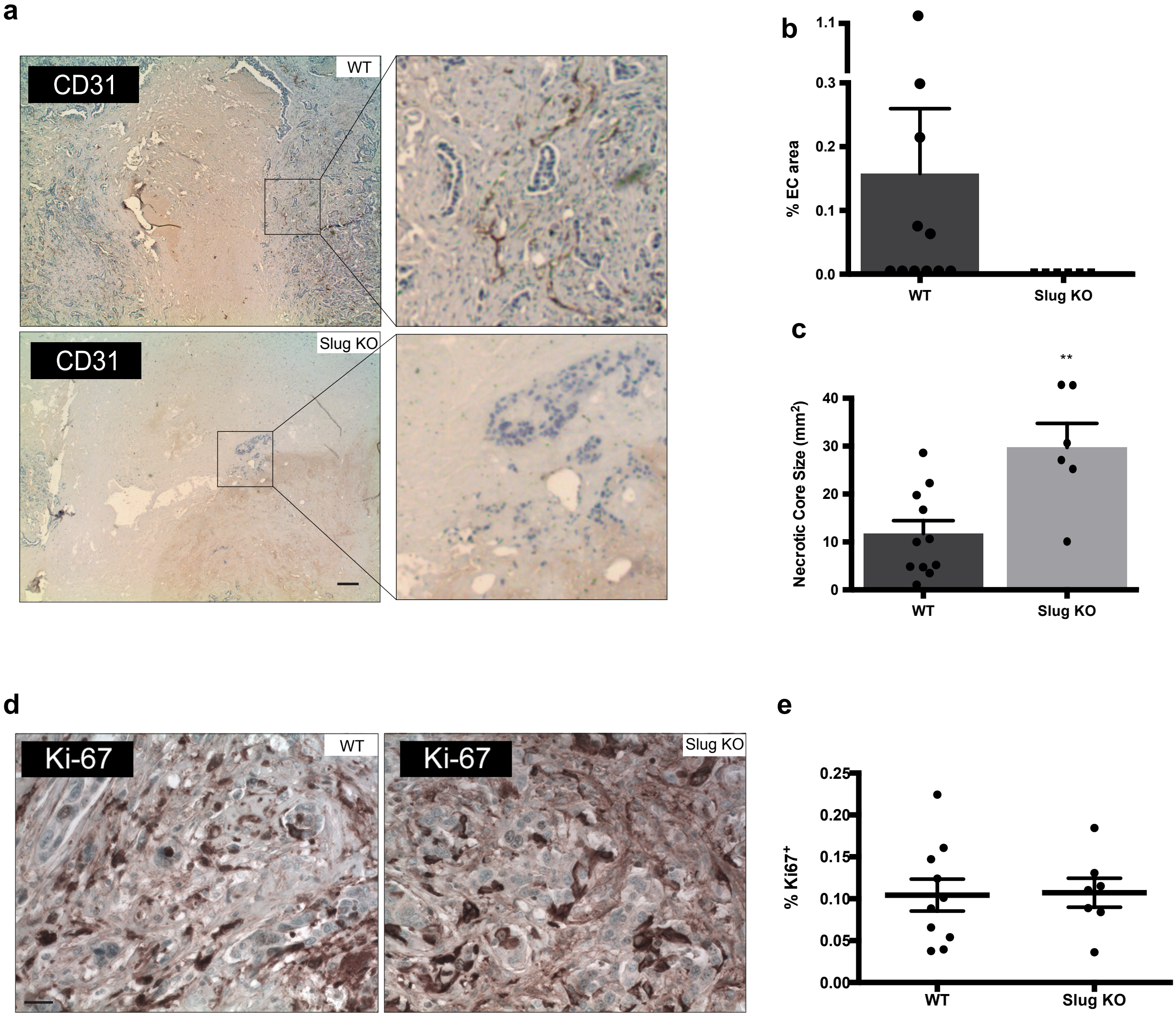

Supplementary Figure 3: Immunohistochemistry analysis of CMT-93 tumors grown in WT vs. SlugKO mice. a) CD31 staining in the core regions of CMT-93 tumors from WT and SlugKO mice. Nuclei were counter-stained with hematoxylin. Scale bar: 200 µm. b) Average percentage of CD31+ areas in the center of CMT-93 tumors from WT (n=11) and SlugKO (n=10) mice. c) Average size of necrotic core areas in CMT-93 tumors from WT (n=11) and SlugKO (n=12) mice. p=0.0035. d) Ki67 staining in CMT-93 tumors from WT and SlugKO mice. Nuclei were counter-stained with hematoxylin. Scale bar: 25 µm. e) Quantitation of Ki67+ nuclei in CMT-93 tumor from WT (n=10) and SlugKO (n=7) mice. Data represent mean ± SEM. Two-tailed unpaired equal-variance t-test. *p<0.05, **p<0.01, ***p<0.005. Source data are provided as a Source Data file.

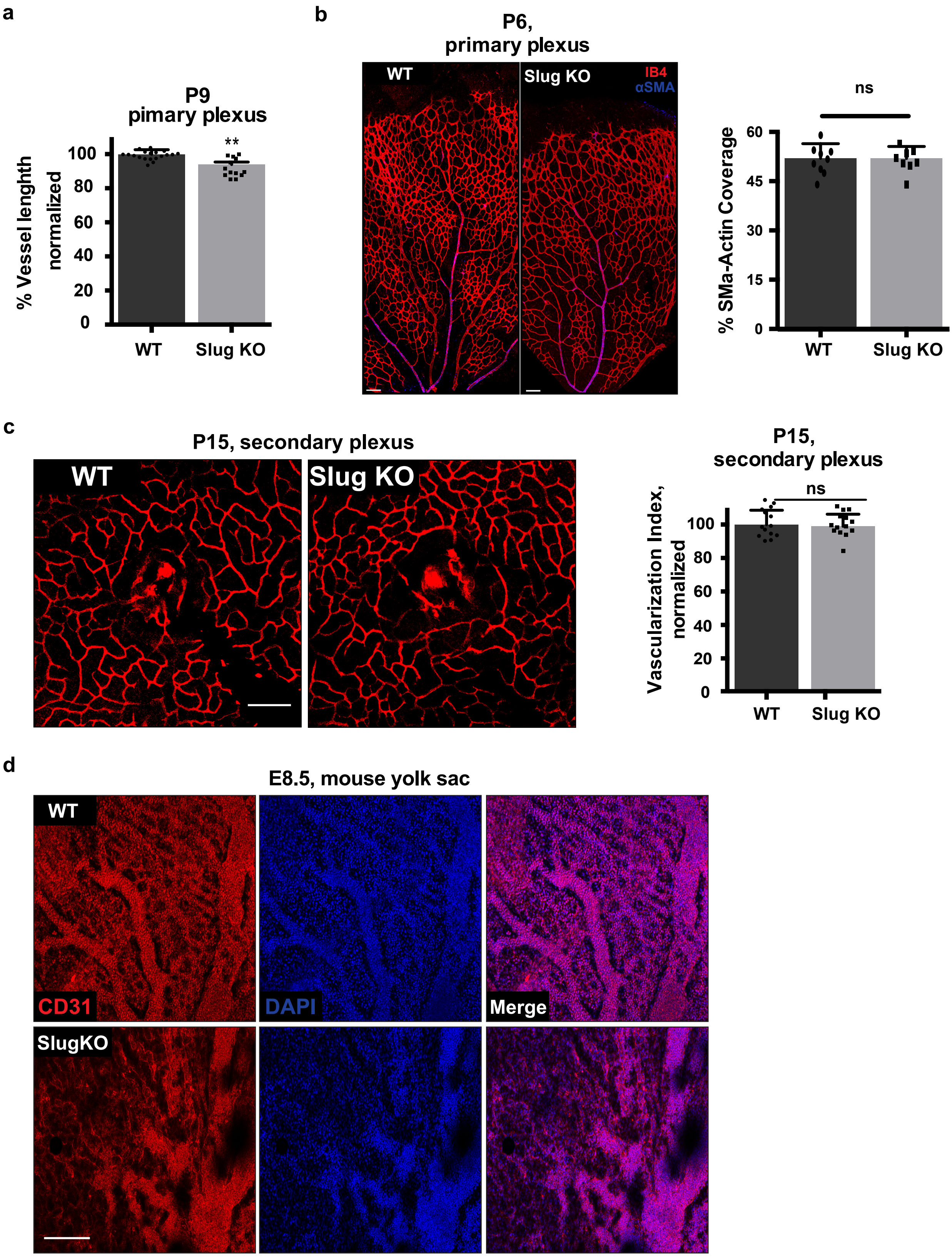

Supplementary Figure 4: Developmental angiogenesis defects in SlugKO mice. a) Average percentage vessel length of retinal leaflet length for secondary plexus in P9 WT (n=19) and SlugKO (n=13) mice. p=0.002. b) Left: IB4 and αSMA staining in P6 WT and SlugKO mouse retina. Scale bar: 100 µm. Right: Average vessel length positive for αSMA staining in WT (n=9) and SlugKO (n=8) mice. c) Left: IB4 staining of secondary plexus in P15 WT and SlugKO mice. Scale bar: 100 µm. Right: Average percentage vessel density of total retinal area for secondary plexus in P15 WT (n=14) and SlugKO (n=14) mice. d) CD31 and DAPI staining of embryonic day 8.5 (E8.5) showing difference in vascular morphology in WT and SlugKO mouse yolk sacs. Scale bar: 100 µm. Data represent mean ± SEM. Two-tailed unpaired equal-variance t-test. *$p<0.05$, **$p<0.01$, ***$p<0.005$. Source data are provided as a Source Data file.

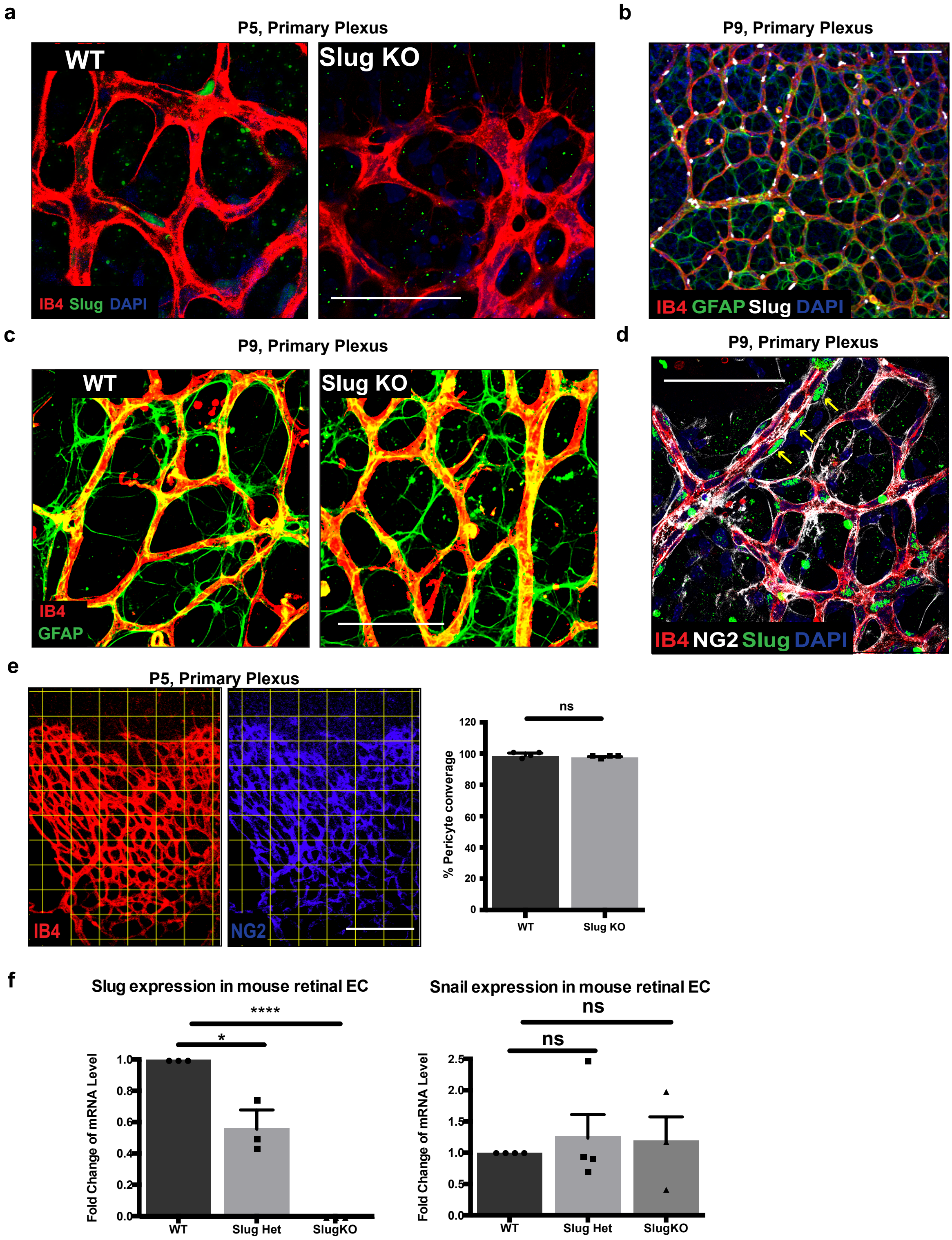

Supplementary Figure 5: Slug expression in retinal cells. a) IB4, Slug and DAPI staining of primary plexus of WT and SlugKO P5 retina showing Slug expression in retinal EC. Scale bar: 100 µm. b) IB4, GFAP, Slug and DAPI staining of primary plexus in WT P9 retina showing Slug expression in retinal EC but not GFAP+ astrocytes. Scale bar: 100 µm. c) IB4 and GFAP staining of primary plexus of WT and SlugKO mice at P9, showing no difference in GFAP+ astrocyte distribution. Scale bar: 100 µm. d) IB4, NG2, Slug and DAPI staining of primary plexus in WT mouse at P9 showing Slug expression in NG2+ pericytes. Scale bar: 100 µm. e) Left: IB4 and NG2 staining images with overlaid grid showing method of pericyte coverage quantification. Scale bar: 100 µm. Right: Average percentage pericyte coverage in P5 WT and SlugKO mouse retinas showing no difference. f) Fold change in Slug and Snail mRNA level in sorted retinal EC from WT (n=3 animals), Slug Het (n=3 animals) and SlugKO (n=3 animals) mice. WT vs. SlugHet p=0.01. WT vs. SlugKO p<0.0001. Data represent mean ± SEM. Two-tailed unpaired equal-variance t-test. *p<0.05, **p<0.01, ***p<0.005. Source data are provided as a Source Data file.

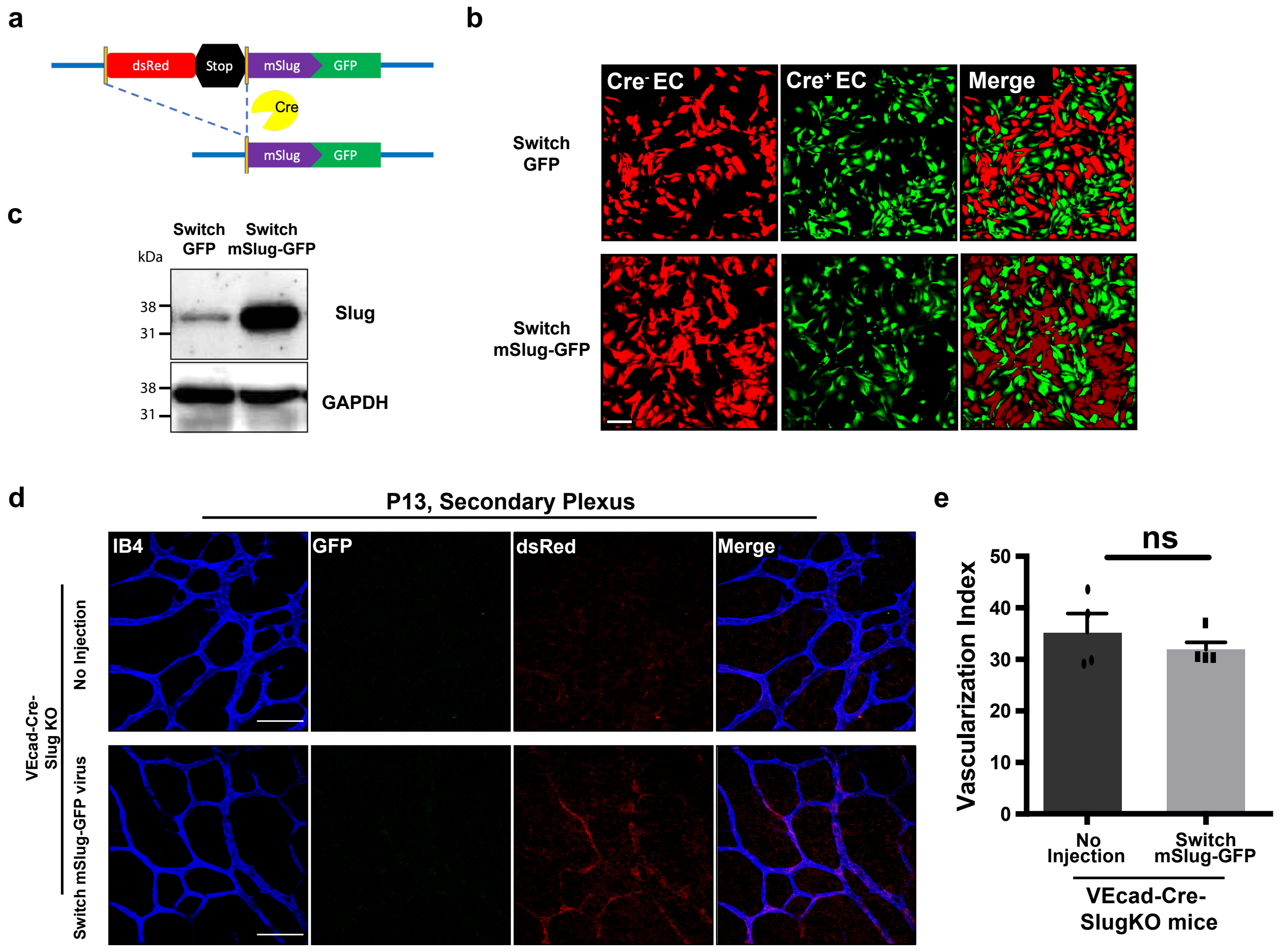

Supplementary Figure 6: In vivo lentiviral-mediated EC-specific Slug expression improves retinal vascularization in SlugKO mice. a) Schematics for Switch virus used in EC-specific rescue experiment. b) GFP and dsRed expression in Cre– and Cre+ EC transduced with titer-matched Switch-GFP or Switch-mSlug-GFP virus. No difference in GFP expression level was observed. Scale bar: 100 μm. c) Western blot showing difference in Slug expression in Cre+ EC transduced with either Switch-GFP or Switch-mSlug-GFP virus. This experiment was conducted once. d) IB4, GFP and dsRed expression in retina of either no-injection control or VEcad-Cre–, SlugKO mice injected with Switch-mSlug-GFP virus. Scale bar: 100 μm. e) Average vascularization index in either no-injection control (n=4) or VEcad-Cre–, SlugKO mice injected with Switch-mSlug-GFP virus (n=4). Data represent mean ± SEM. Two-tailed unpaired equal-variance t-test. *p<0.05, **p<0.01, ***p<0.005. Source data are provided as a Source Data file.

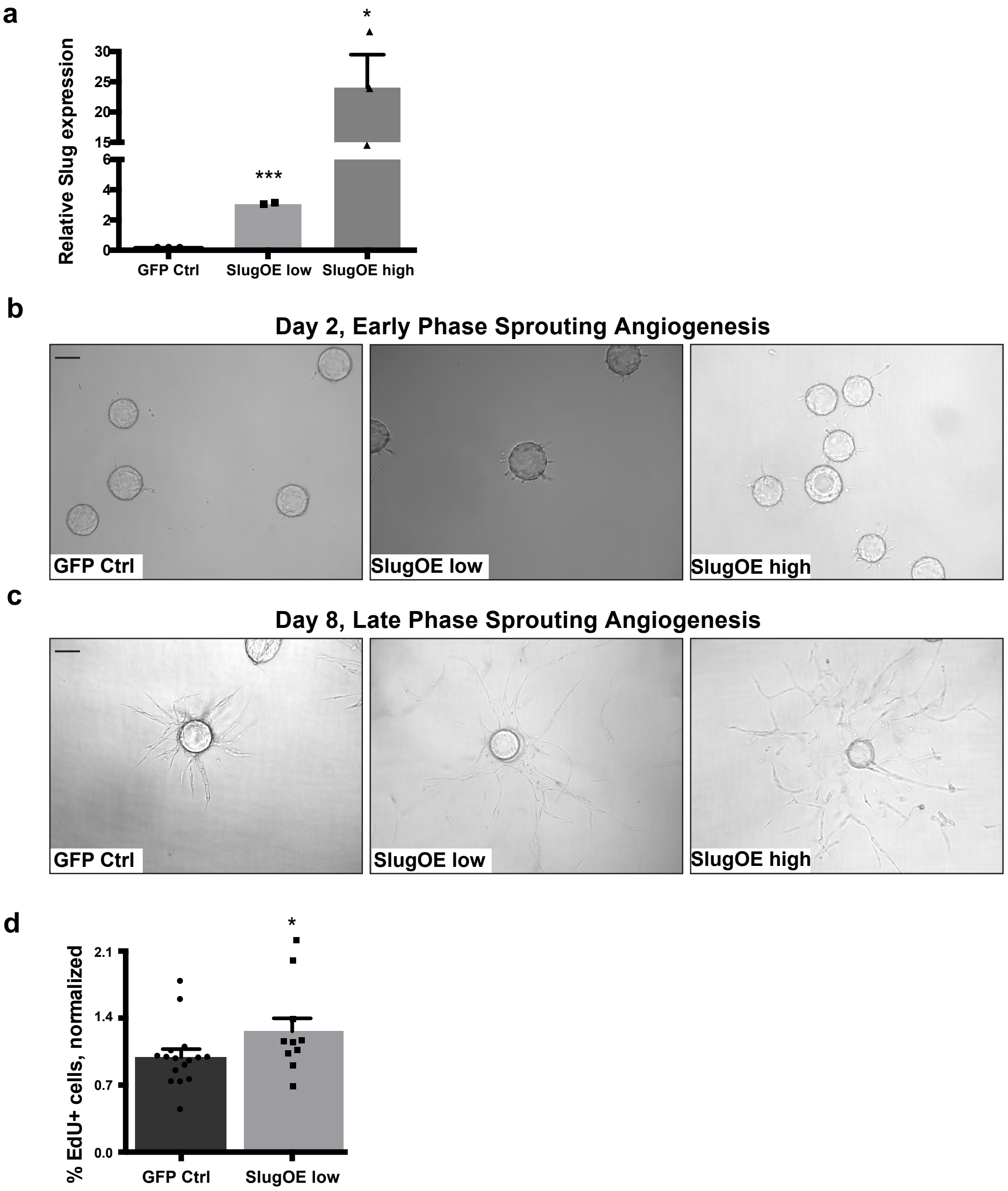

Supplementary Figure 7: Defective in vitro vascular morphogenesis. a) qPCR analysis of different levels of Slug overexpression in transduced SlugOE cells (SlugOE low n=2 cell lines, p<0.0001. SlugOE high n=3 cell lines. p=0.012) compared to GFP control (n=3 cell lines). Two-tailed unpaired equal-variance t-test. b) Low magnification view of the GFP, SlugOElow, SlugOEhigh beads at day 2 (early phase sprouting angiogenesis) of the fibrin-gel bead assay. c) Low magnification view of the GFP, SlugOElow, SlugOEhigh beads at day 8 (late phase sprouting angiogenesis) of the fibrin-gel bead assay. d) Average percentage EdU+ nuclei per view in GFP control (n=16 devices) and SlugOElow (n=10 devices) EC. One-tailed unpaired equal-variance t-test. p=0.043. Data represent mean ± SEM. *p<0.05, **p<0.01, ***p<0.005. Source data are provided as a Source Data file.

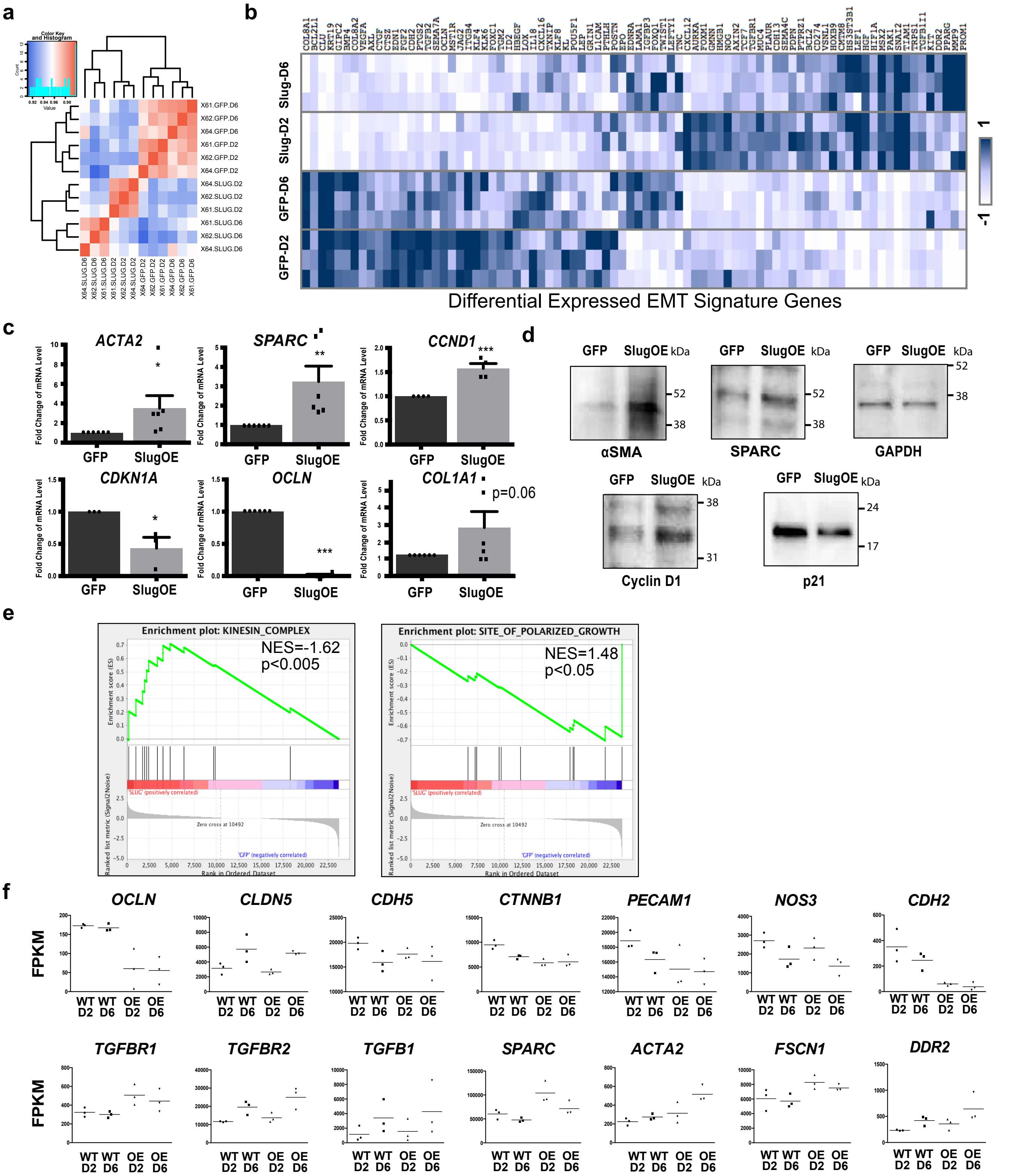

Supplementary Figure 8: SlugOE bead assay RNA-seq analysis. a) RNA-seq analysis of gene expression in GFP control and SlugOE EC from day 2 and day 6 of the fibrin-gel bead assay showing high concordance among biological replicates. b) Differentially expressed EMT signature genes. c) qPCR results showing average fold change in expression of genesinvolved in EMT in GFP (n=3 cell lines) and SlugOE (n=3 cell lines) EC. ACTA2 p=0.04, SPARC p=0.0095, CCND1 p=0.0007, CDKN1A p=0.014, OCLN p<0.0001. d) Western blot of proteins involved in EMT in GFP control and SlugOE EC. This experiment was conducted twice. e) GSEA analysis shows enrichment in the cell division program and reduced polarization in SlugOE EC. f) Average FPKM values of genes involved in cell-cell junctions, TGFβ signaling, EMT and normal EC functions (n=3 cell lines for each condition). Data represent mean ± SEM. One-tailed unpaired equal-variance t-test. *p<0.05, **p<0.01, ***p<0.005. Source data are provided as a Source Data file.

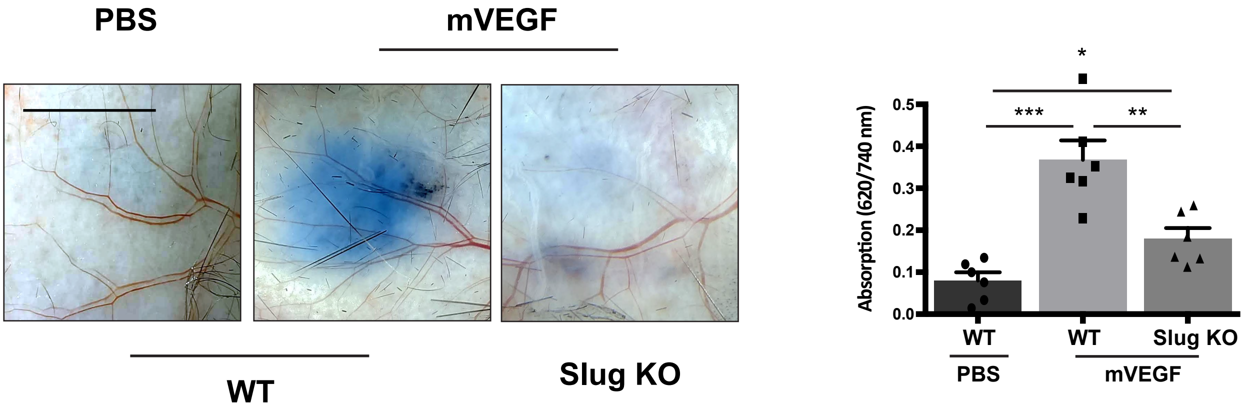

Supplementary Figure 9: Miles in vivo permeability assay. Left: images from Miles Assay showing difference in Evans Blue dye leakage in WT and SlugKO mice with local injection of PBS or mVEGF. Scale bar: 5mm. Right: Average absorption at 620/740nm of skin samples from WT (n=6 for both conditions, p=0.0002.) and SlugKO mice (n=6 animals, p=0.01.) injected with PBS and mVEGF. Data represent mean ± SEM. Two-tailed unpaired equal-variance t-test. *p<0.05, **p<0.01, ***p<0.005. Source data are provided as a Source Data file.

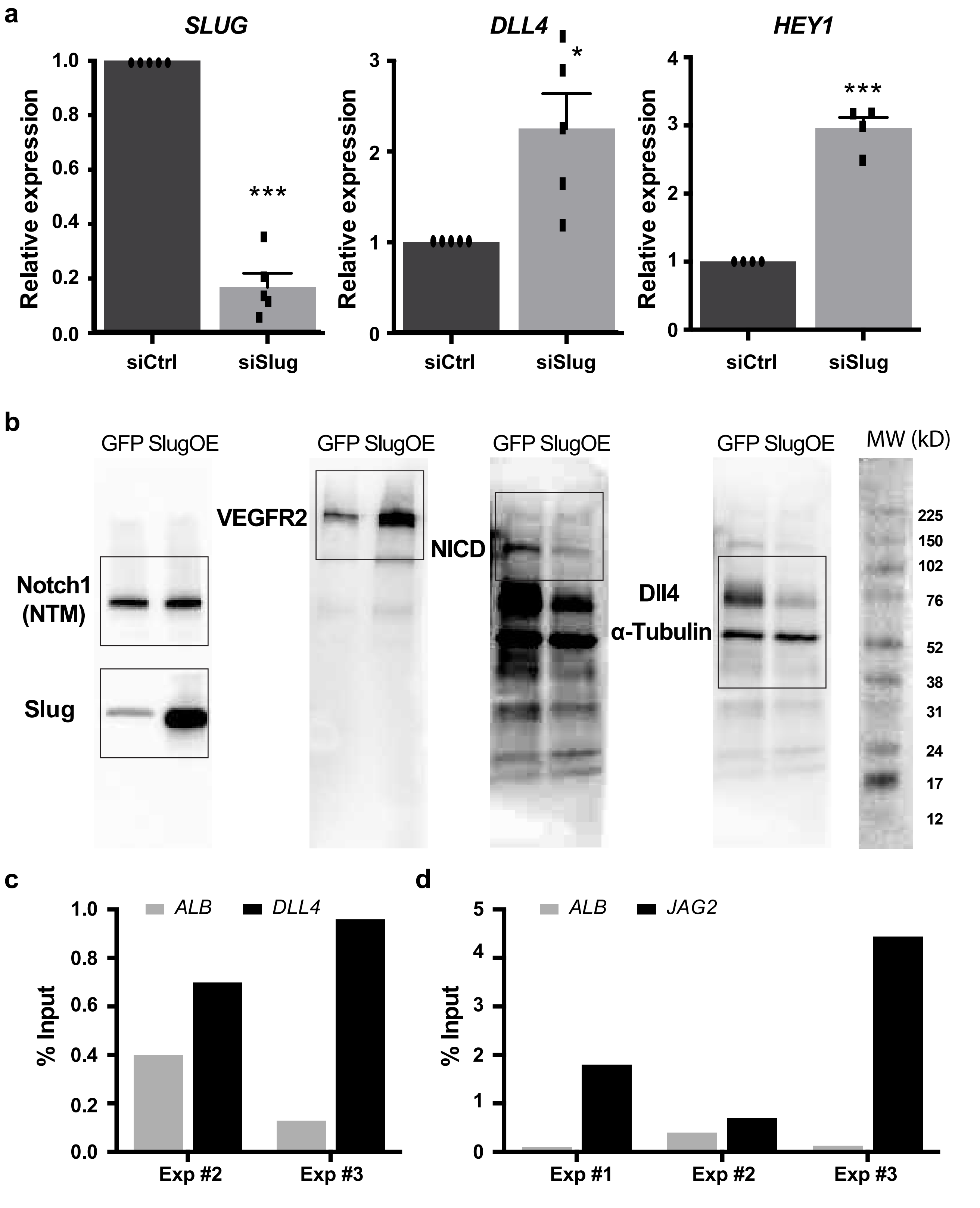

Supplementary Figure 10: RNA-seq Notch pathway validation. a) qPCR analysis of Slug and its target genes in Notch pathway upon knockdown of endogenous Slug using a second siRNA. *SLUG* $p<0.0001$, *DLL4* $p=0.012$, *HEY1* $p<0.0001$. b) Full western blots for Notch-related proteins in GFP control and SlugOE HUVEC. c) ChIP-qPCR showing enriched Slug binding to DLL4 promoter in SlugOE EC, experiment #2 & #3. d) ChIP-qPCR showing enriched Slug binding to JAG2 promoter in SlugOE EC, 3 experiments. Data represent mean ± SEM. Two-tailed unpaired equal-variance t-test. *$p<0.05$, **$p<0.01$, ***$p<0.005$. Source data are provided as a Source Data file.

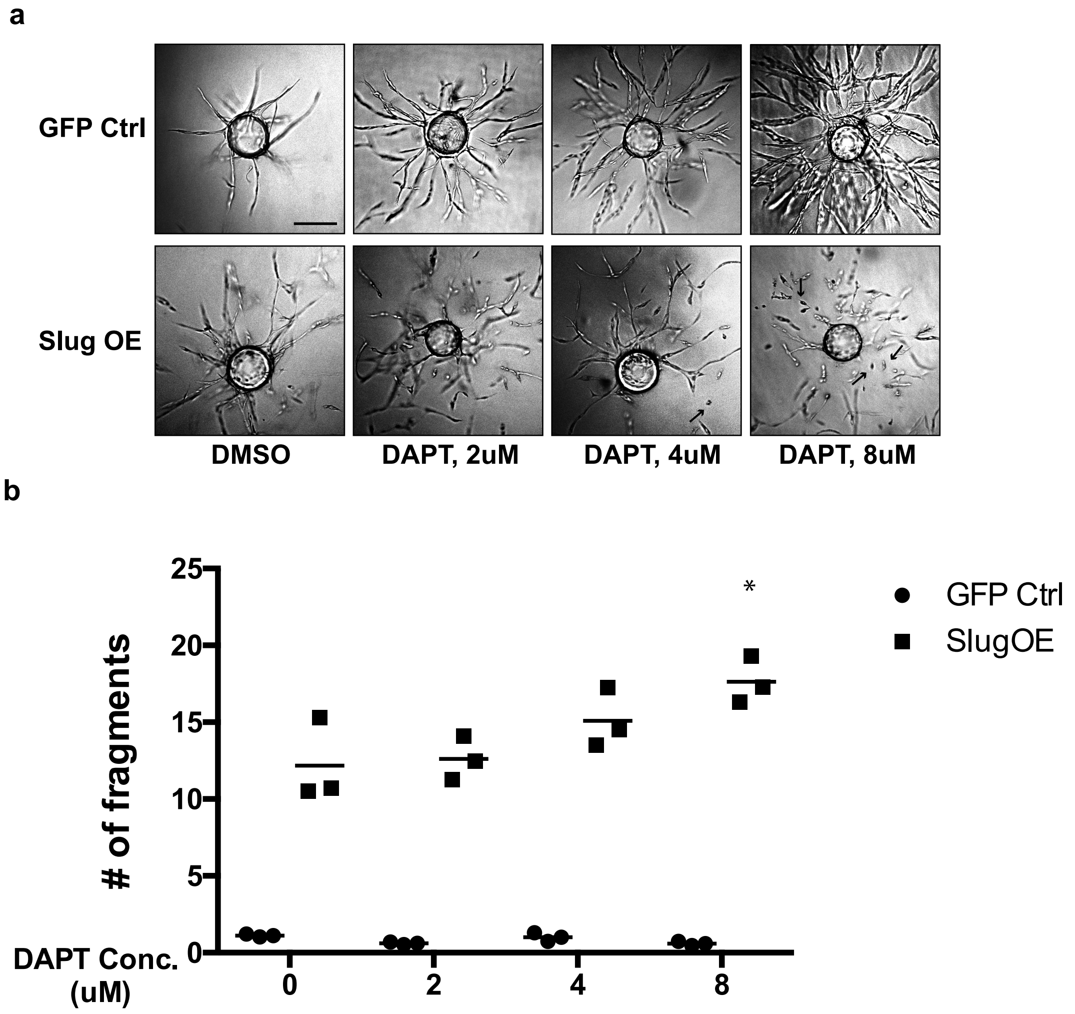

Supplementary Figure 11: Notch-VEGF pathway interactions. a) DAPT treatment in the fibrin-gel bead assay leads to increased fragmentation and higher levels of EndoMT in SlugOE EC. Black arrow: individual EC that have lost polarity completely and appear round. Scale bar: 100 µm. b) Number of disconnected fragments in SlugOE EC increases in a dose-dependent manner following DAPT treatment (n=3 cell lines. P=0.017). Data represent mean ± SEM. Two-tailed unpaired equal-variance t-test. *p<0.05, **p<0.01, ***p<0.005. Source data are provided as a Source Data file.

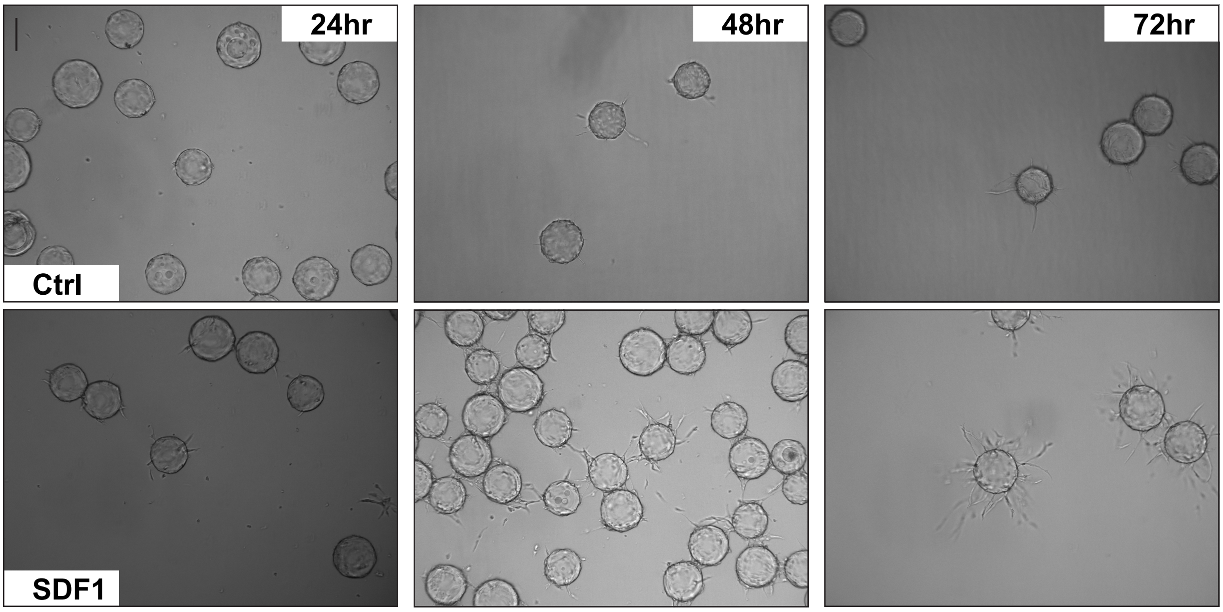

Supplementary Figure 12: SDF1α promotes in-vitro angiogenesis (zoomed out). Low magnification view of the fibrin-gel bead assay treated with control and SDF1α over 72 hours. Scale bar: 100 μm. Source data are provided as a Source Data file.

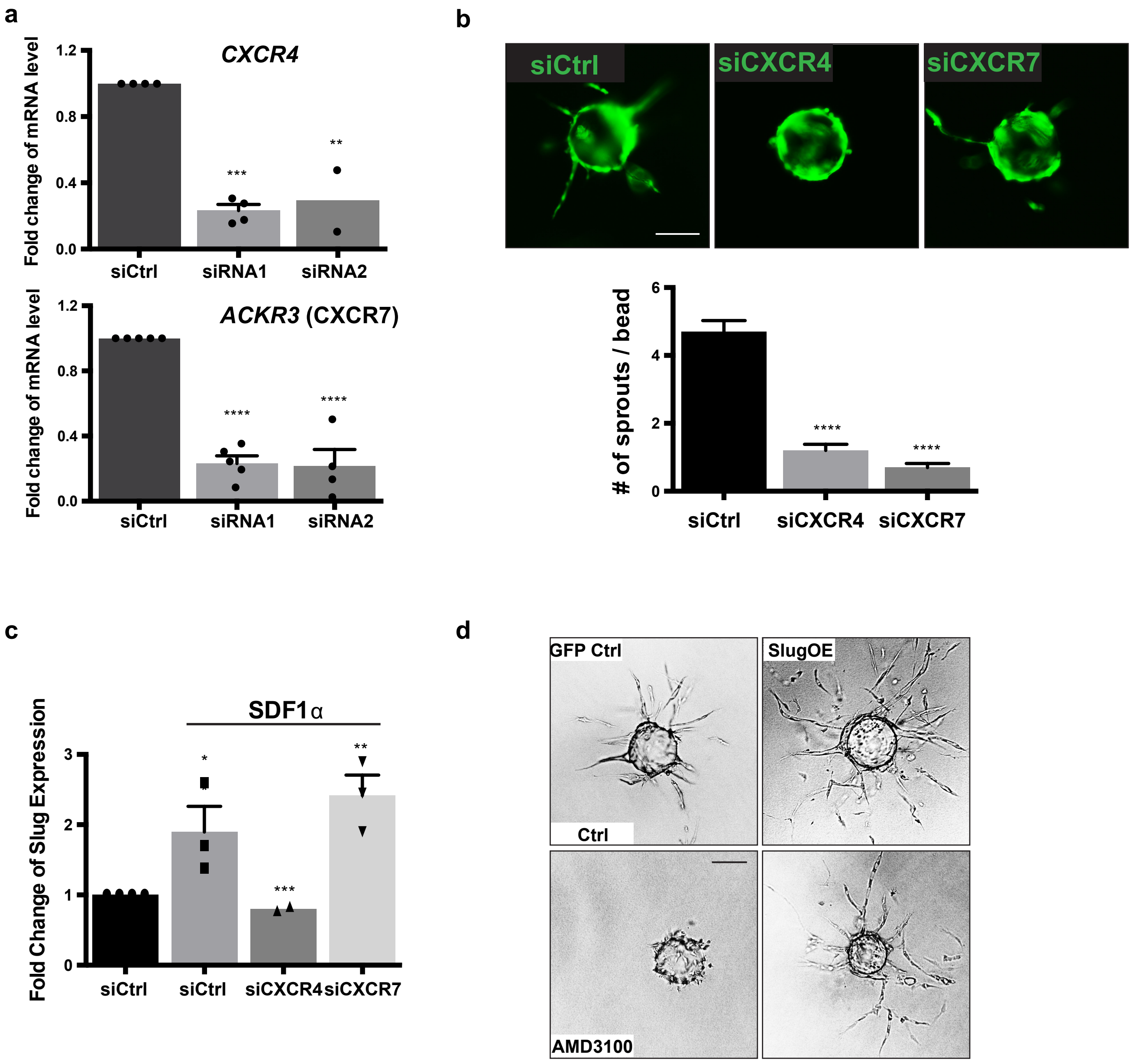

Supplementary Figure 13: SDF1α -CXCR4 signaling promotes angiogenic sprouting by induction of Slug. a) qPCR analysis showing successful CXCR4 (siRNA1 n=4 cell lines, p=0.003. siRNA2 n=2 cell lines, p=0.0004.) and CXCR7 (siRNA1 n=5 cell lines, p<0.0001. siRNA2 n=4 cell lines, p=0.002.) knockdown using two independent siRNA for each gene . b) Top: Representative images showing knockdown of either CXCR4 or CXCR7 leads to reduced sprouting in the fibrin-gel bead assay (n=20 beads for all conditions). Image taken on day 5 of the assay. Scale bar: 100 μm. Bottom: Average number of sprouts per bead with siControl, siCXCR4 (p<0.0001) and siCXCR7 (p<0.0001) EC. c) qPCR analysis showing SDF1α induction of Slug expression with a second, independent set of siControl (n=4 cell lins, p=0.03), siCXCR4 (n=2 cell lines, p=0.0004) and siCXCR7 (n=3 cell lines, p=0.002). d) Fibrin-gel bead assay images showing rescue of AMD3100-mediated inhibition of sprouting by Slug overexpression. Scale bar: 100 μm. Data represent mean ± SEM. Two-tailed unpaired equal-variance t-test. *p<0.05, **p<0.01, ***p<0.005. Source data are provided as a Source Data file.

a

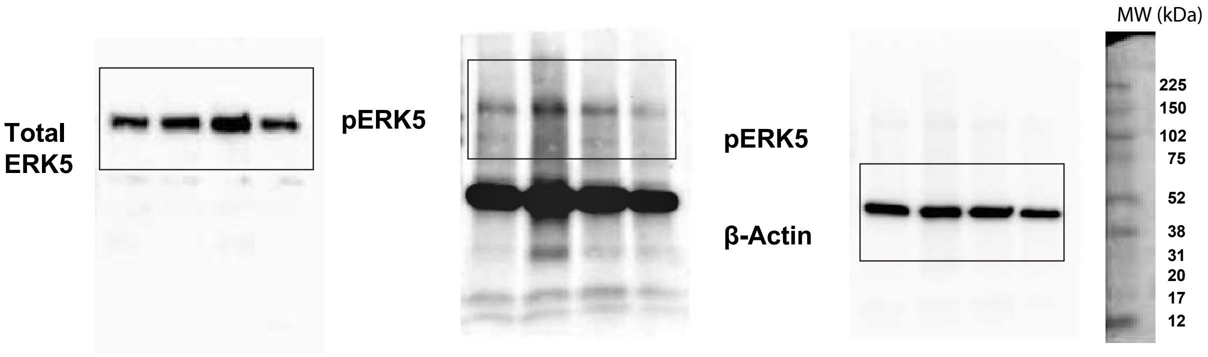

b

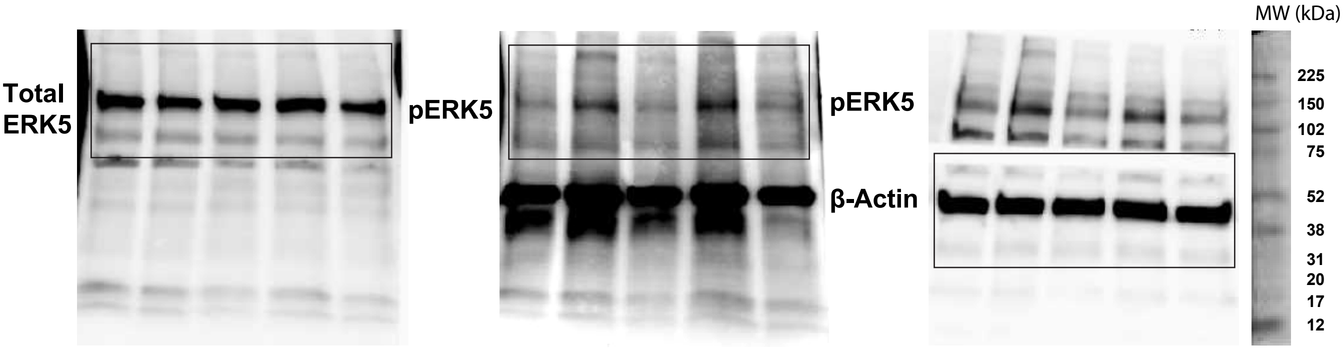

Supplementary Figure 14: SDF1α-CXCR4 induction of ERK5 phosphorylation, whole blots. a) Full western blots of ERK5 phosphorylation in inhibitor experiment shown in Fig. 7G. b) Full western blots of ERK5 phosphorylation in siCXCR4/7 experiment shown in Fig.7H. Source data are provided as a Source Data file.

Supplementary Table 1: Human Q-PCR Primer Sequences

Primer Name	Sequence
human *SNAI2* forward	5’-AGATGCATATTCGGACCCAG-3’
human *SNAI2* reverse	5’-CCTCATGTTTGTGCAGGAGA-3’
human *SNAI1* forward	5'-GCCTTCAACTGCAAATACTGC-3'
human *SNAI1* reverse	5'-CTTCTTGACATCTGAGTGGGT-3'
human *HEY1* forward	5’-TGGATCACCTGAAAATGCTG-3’
human *HEY1* reverse	5'-CGAAATCCCAAACTCCGATA-3'
human *DLL4* forward	5'-GTAACGAATGCATCCCCCACAAT-3'
human *DLL4* reverse	5'-CTCCCCAGCCCTCATCACAAGTA-3'
human *KDR* forward	5'-GGAAGCTCCTGAAGATCTGT-3'
human *KDR* reverse	5'-GAGGATATTTCGTGCCGC-3'
human *ACTA2* forward	5'-CAAGGCCAACCCGGGAGAAAAT-3'
human *ACTA2* reverse	5'-ACCGCCTGGATAGCCACATAC-3'
human *SPARC* forward	5'-CAGGGCTCTTCTCAGGGGCTCTA-3'
human *SPARC* reverse	5'-CAACCGATTCACCAACTCCACTTT-3'
human *CCND1* forward	5'-GCTGCGAAGTGGAAACCATC-3'
human *CCND1* reverse	5'-CCTCCTTCTGCACACATTTGAA-3'
human *CDKN1A* forward	5'-TGTCCGTCAGAACCCATGC-3'
human *CDKN1A* reverse	5'-AAAGTCGAAGTTCCATCGCTC-3'
human *OCLN* forward	5'-ACAAGCGGTTTTATCCAGAGTC-3'
human *OCLN* reverse	5'-GTCATCCACAGGCGAAGTTAAT-3'
human *COL1A1* forward	5'-CGATGGCTGCACGAGTCACAC-3'
human *COL1A1* reverse	5'-GGGCAGGCGGGAGGTCTT-3'
human *CXCR4* forward	5’- AATCTTCCTGCCCACCATCTACTCC-3’
human *CXCR4* reverse	5’- GCGGTCACAGATATATCTGTCATCTGCC-3’
human *ACKR3* forward	5’- ACGTGGTGGTCTTCCTTGTC-3’
human *ACKR3* reverse	5’- AAGGCCTTCATCAGCTCGTA-3’
human *GAPDH* forward	5'-GAGTCAACGGATTTGGTCGT-3'
human *GAPDH* reverse	5'-TTGATTTTGGAGGGATCTCG-3'
ChIP human *DLL4* forward	5'-TCCTCGGCGCGGTCG-3'
ChIP human *DLL4* reverse	5'-GCTCCCCTTTGGCCTTCTC-3'
ChIP human *JAG2* forward	5'-GCCCTCCACCGCCTTTA-3'
ChIP human *JAG2* reverse	5'-GGTGAAGGCAGCGGGTC-3'
ChIP human *ALB* forward	5'-ACATTGACAAGGTCTTGTGGAGA-3'
ChIP human *ALB* reverse	5'-TGGCTGCCAACCGATTACAA-3'

Supplementary Table 2: Mouse Q-PCR Primer Sequences

mouse *Snai2* forward	5'-CACTCCACTCTCCTTTACC-3'
mouse *Snai2* reverse	5'-CAGACTCCTCATGTTTATGC-3'
mouse *Snai1* forward	5'-CTGCTTCGAGCCATAGAACTAAAG-3'
mouse *Snai1* reverse	5'-GAGGGGAACTATTGCATAGTCTGT-3'
mouse *Gapdh* forward	5'-AGGTCGGTGTGAACGGATTTG-3'
mouse *Gapdh* reverse	5'-TGTAGACCATGTAGTTGAGGTCA-3'
