# Supplementary material for: Slug regulates the Dll4-Notch-VEGFR2 axis to control endothelial cell activation and angiogenesis
Source: Nat Commun. 2020 Oct 26;11:5400. doi: 10.1038/s41467-020-18633-z (PMC7588439; doi:10.1038/s41467-020-18633-z)
Supplement: Supplementary file 3 — Description of Additional Supplementary Files [file 41467_2020_18633_MOESM3_ESM.docx]

Description of Additional Supplementary Files

Title: Supplementary Dataset 1:

Description: Differential Expression Gene Analysis. Differential expression gene analysis conducted between each pair of the four experimental groups in the RNA-seq analysis: GFP D2 vs. SlugOE D2, GFP D6 vs. SlugOE D6, GFP D2 vs. GFP D2, SlugOE D2 vs. SlugOE D6.
