# Supplementary material for: Slug regulates the Dll4-Notch-VEGFR2 axis to control endothelial cell activation and angiogenesis
Source: Nat Commun. 2020 Oct 26;11:5400. doi: 10.1038/s41467-020-18633-z (PMC7588439; doi:10.1038/s41467-020-18633-z)
Supplement: Supplementary file 5 — Reporting Summary [file 41467_2020_18633_MOESM5_ESM.pdf]

nature research

Corresponding author(s): Christopher Hughes

Last updated by author(s): Jul 20, 2020

Reporting Summary

Nature Research wishes to improve the reproducibility of the work that we publish. This form provides structure for consistency and transparency in reporting. For further information on Nature Research policies, see Authors & Referees and the Editorial Policy Checklist .

Statistics

For all statistical analyses, confirm that the following items are present in the figure legend, table legend, main text, or Methods section.

n/a	Confirmed	
☐	☒	The exact sample size (*n*) for each experimental group/condition, given as a discrete number and unit of measurement
☐	☒	A statement on whether measurements were taken from distinct samples or whether the same sample was measured repeatedly
☐	☒	The statistical test(s) used AND whether they are one- or two-sided<br>*Only common tests should be described solely by name; describe more complex techniques in the Methods section.*
☐	☒	A description of all covariates tested
☐	☒	A description of any assumptions or corrections, such as tests of normality and adjustment for multiple comparisons
☐	☒	A full description of the statistical parameters including central tendency (e.g. means) or other basic estimates (e.g. regression coefficient) AND variation (e.g. standard deviation) or associated estimates of uncertainty (e.g. confidence intervals)
☐	☒	For null hypothesis testing, the test statistic (e.g. *F*, *t*, *r*) with confidence intervals, effect sizes, degrees of freedom and *P* value noted<br>*Give P values as exact values whenever suitable.*
☒	☐	For Bayesian analysis, information on the choice of priors and Markov chain Monte Carlo settings
☒	☐	For hierarchical and complex designs, identification of the appropriate level for tests and full reporting of outcomes
☒	☐	Estimates of effect sizes (e.g. Cohen's *d*, Pearson's *r*), indicating how they were calculated

Our web collection on statistics for biologists contains articles on many of the points above.

Software and code

Policy information about availability of computer code

Data collection	Image Lab v.3.0.1, Quantity One v.4.6.6, Nanodrop 2000/2000c, SPOT v.5.1.23, LAS X v.3.5.5.19976, BD FACSDiva™,
Data analysis	FASTQC(v. 0.11.2), Trimmomatic(v.0.32), Tophat2 (v.2.0.12), Bowtie2 (v.2.2.3), Samtools (v.0.1.19), Cufflinks (v.2.1.1), HTSeq (v.0.6.1p1.), DESeq2, GSEA, Metascape, R, JavaTree, ImageJ 1.48v, Photoshop CC2015 (Adobe), Graphpad Prism 6

For manuscripts utilizing custom algorithms or software that are central to the research but not yet described in published literature, software must be made available to editors/reviewers. We strongly encourage code deposition in a community repository (e.g. GitHub). See the Nature Research guidelines for submitting code & software for further information.

Data

Policy information about availability of data

All manuscripts must include a data availability statement. This statement should provide the following information, where applicable:

- Accession codes, unique identifiers, or web links for publicly available datasets
- A list of figures that have associated raw data
- A description of any restrictions on data availability

All data generated or analyzed during this study are included in this published article (and its supplementary information files). The results of differential-gene-expression analysis results from the RNA-seq experiment are included in the supplemental table 1. Raw data is deposited into the GEO database under the accession code GSE154546. The source data underlying Figures 1A, B, E, F, H, 2B, D, E, F, H K, 3D-G, 5B-H, 6C, D, 7B, D, E-I, and Supplementary Figures 1a, b, 2, 3b, e, 4a-c, 5e, f, 6c, e, 7a, d, 8c, d, 9, 10a-d, 11b, 13a-c, 14a, b are provided as a Source Data File.

Field-specific reporting

Please select the one below that is the best fit for your research. If you are not sure, read the appropriate sections before making your selection.

☒ Life sciences ☐ Behavioural & social sciences ☐ Ecological, evolutionary & environmental sciences

For a reference copy of the document with all sections, see nature.com/documents/nr-reporting-summary-flat.pdf

Life sciences study design

All studies must disclose on these points even when the disclosure is negative.

Sample size	Sample size calculation was not performed. Sample size was chosen based on similar studies performed previously. In particular, sample size for the tumor study can be referenced from Villarejo A, Molina-Ortiz P, Montenegro Y, et al. Loss of Snail2 favors skin tumor progression by promoting the recruitment of myeloid progenitors. Carcinogenesis. 2015;36(5):585-597. doi:10.1093/carcin/bgv021. Sample size for the retina study can be referenced from Suchting S, Freitas C, le Noble F, et al. The Notch ligand Delta-like 4 negatively regulates endothelial tip cell formation and vessel branching. Proc Natl Acad Sci U S A. 2007;104(9):3225-3230. doi:10.1073/pnas.0611177104. Sample size for bead assay can be referenced from Welch-Reardon KM, Ehsan SM, Wang K, et al. Angiogenic sprouting is regulated by endothelial cell expression of Slug. J Cell Sci. 2014;127(Pt 9):2017-2028. doi:10.1242/jcs.143420. Sample size for the VMO device can be derived from Phan DTT, Wang X, Craver BM, et al. A vascularized and perfused organ-on-a-chip platform for large-scale drug screening applications. Lab Chip. 2017;17 (3):511-520. doi:10.1039/c6lc01422d. Sample size for the RNA-seq and ChIP experiment was referenced from Mistry DS, Chen Y, Wang Y, Zhang K, Sen GL. SNAI2 controls the undifferentiated state of human epidermal progenitor cells. Stem Cells. 2014;32(12):3209-3218. doi:10.1002/stem.1809.
Data exclusions	No data was excluded from analysis.
Replication	All experiments were replicated on multiple cell lines and animals and the replications were always done indepdently. All replications were successful and included for data analysis.
Randomization	All cells and animals used were randomly assigned into either control or experimental groups. All cells and animals tissue materials were collected in random order after experiments/treatments. If, under any circumstances where sample allocation cannot be randomized during treatment, special attention is given to ensure that sample harvest and/or processing is randomized.
Blinding	For quantifications, the researchers were blinded to experimental conditions.

Reporting for specific materials, systems and methods

We require information from authors about some types of materials, experimental systems and methods used in many studies. Here, indicate whether each material, system or method listed is relevant to your study. If you are not sure if a list item applies to your research, read the appropriate section before selecting a response.

Materials & experimental systems

n/a	Involved in the study	
☐	☒	Antibodies
☐	☒	Eukaryotic cell lines
☒	☐	Palaeontology
☐	☒	Animals and other organisms
☒	☐	Human research participants
☒	☐	Clinical data

Methods

n/a	Involved in the study	
☒	☐	ChIP-seq
☐	☒	Flow cytometry
☒	☐	MRI-based neuroimaging

Antibodies

Antibodies used	fluorescein/Rhodamine/Alexa647 labeled GSL I isolectin B4 (1:500, Vector), rabbit anti-Slug (1:100, Cell Signaling 9585), mouse anti-NG2 (1:100, Millipore 5384), mouse anti-GFAP (1:100, Millipore 360), mouse anti-rabbit anti-Claudin5 (1:400, Abcam 53765), rabbit anti-VE-cadherin (1:400, Enzo ALX-210-232), mouse anti-CD31 (1:50, Dako 0823), goat anti-Dll4 (1:50, R&D AF1389), rabbit anti-VEGFR2 (1:100, Cell Signaling 2479), goat anti-rabbit-A488 (1:500, Invitrogen A11034), goat anti-rabbit-A594 (1:500, Invitrogen R37117), goat anti-mouse-A647 (1:500, Invitrogen A21235), donkey anti-goat-A647 (1:500, Invitrogen A21447). Antibodies for western blot: mouse anti-alpha-SMA (1:1000, Dako M0851), rabbit anti-SPARC (1:1000, Cell Signaling 8725), mouse anti-CCND1 (1:1000, Santa Cruz Biotech, sc-8396), rabbit anti-p21 (1:1000, Cell Signaling 2947), rabbit anti-NICD (1:1000, Cell Signaling 4147), rabbit anti-Notch1 XP (1:1000, Cell Signaling 3608), rabbit anti-Dll4 (1:1000, Cell Signaling 2589), rabbit anti-Slug (1:500, Cell Signaling, 9585), rabbit anti-VEGFR2 (1:1000, Cell Signaling 2479), rabbit anti-Hey1 (1:500, Abcam 22614), rabbit anti-pERK5 (1:1000, Cell Signaling 3371), rabbit anti-ERK5 (1:1000, Cell Signaling 3552), rabbit anti-alpha-Tubulin (1:2000, Cell Signaling 2144), rabbit anti-GAPDH (1:2000, Cell Signaling 5174), rabbit anti-beta-Actin-HRP (1:2000, Cell Signaling 5125), anti-rabbit-HRP (1:2000, Cell Signaling 7074), goat anti-mouse-HRP (1:2000, Santa Cruz Biotech sc-2031). Antibody for FACS sorting: rat anti-mouse-CD31 (1:200, Dianova, DIA-310), goat anti-rat-A488 (1:500, Invitrogen, A-11006). Antibody for

immunohistochemistry: rat anti-mouse-CD31 (1:75, Dianova, DIA-310), mouse anti-Ki67 (Roche/Venatana 790-4286). Antibody for ChIP: rabbit anti-Slug (1:50, Santa Cruz Biotech 15391).

Validation

All antibodies used in this study were commercially available. All antibodies were either validated by the manufacturer or by other users as indicated by previous publications on the manufacturers website (see detail for primary antibody citations below). Negative controls were used to validate the antibody signal whenever possible.

Rabbit anti-Slug (1:100, Cell Signaling 9585): IF: Guen VJ, Chavarria TE, Kröger C, Ye X, Weinberg RA, Lees JA. EMT programs promote basal mammary stem cell and tumor-initiating cell stemness by inducing primary ciliogenesis and Hedgehog signaling. Proc Natl Acad Sci U S A. 2017;114(49):E10532-E10539. doi:10.1073/pnas.1711534114. WB: Zhang Q, Yan G, Lei J, et al. The SP1-12LOX axis promotes chemoresistance and metastasis of ovarian cancer. Mol Med. 2020;26(1):39. Published 2020 May 6. doi:10.1186/s10020-020-00174-2.

Rabbit anti-VEGFR2 (1:100, Cell Signaling 2479): IF: Gong B, Li Z, Xiao W, et al. Sec14l3 potentiates VEGFR2 signaling to regulate zebrafish vasculogenesis. Nat Commun. 2019;10(1):1606. Published 2019 Apr 8. doi:10.1038/s41467-019-09604-0. WB: Testini C, Smith RO, Jin Y, et al. Myc-dependent endothelial proliferation is controlled by phosphotyrosine 1212 in VEGF receptor-2 [published correction appears in EMBO Rep. 2020 May 6;21(5):e50409]. EMBO Rep. 2019;20(11):e47845. doi:10.15252/embr.201947845

rabbit anti-NICD (1:1000, Cell Signaling 4147): WB: Fu R, Lv WC, Xu Y, et al. Endothelial ZEB1 promotes angiogenesis-dependent bone formation and reverses osteoporosis. Nat Commun. 2020;11(1):460. Published 2020 Jan 23. doi:10.1038/s41467-019-14076-3

rabbit anti-Notch1 XP (1:1000, Cell Signaling 3608), WB: Zhou P, Li Q, Su S, et al. Interleukin 37 Suppresses M1 Macrophage Polarization Through Inhibition of the Notch1 and Nuclear Factor Kappa B Pathways. Front Cell Dev Biol. 2020;8:56. Published 2020 Feb 14. doi:10.3389/fcell.2020.00056

rabbit anti-Dll4 (1:1000, Cell Signaling 2589), WB: Tetzlaff F, Adam MG, Feldner A, et al. MPDZ promotes DLL4-induced Notch signaling during angiogenesis. Elife. 2018;7:e32860. Published 2018 Apr 5. doi:10.7554/eLife.32860

Rabbit anti-SPARC (1:1000, Cell Signaling 8725), WB: Cao T, Jiang Y, Wang Z, et al. H19 lncRNA identified as a master regulator of genes that drive uterine leiomyomas. Oncogene. 2019;38(27):5356-5366. doi:10.1038/s41388-019-0808-4

Rabbit anti-p21 (1:1000, Cell Signaling 2947), WB: Jones GG, Del Río IB, Sari S, et al. SHOC2 phosphatase-dependent RAF dimerization mediates resistance to MEK inhibition in RAS-mutant cancers. Nat Commun. 2019;10(1):2532. Published 2019 Jun 10. doi:10.1038/s41467-019-10367-x

rabbit anti-pERK5 (1:1000, Cell Signaling 3371), WB: Ruiz-Velasco A, Zi M, Hille SS, et al. Targeting mir128-3p alleviates myocardial insulin resistance and prevents ischemia-induced heart failure. Elife. 2020;9:e54298. Published 2020 Mar 30. doi:10.7554/eLife.54298

rabbit anti-ERK5 (1:1000, Cell Signaling 3552), WB: Parascandolo A, Bonavita R, Astaburuaga R, et al. Effect of naive and cancer-educated fibroblasts on colon cancer cell circadian growth rhythm. Cell Death Dis. 2020;11(4):289. Published 2020 Apr 27. doi:10.1038/s41419-020-2468-2

rabbit anti-alpha-Tubulin (1:2000, Cell Signaling 2144), WB: Liao Y, Zhao J, Bulek K, et al. Inflammation mobilizes copper metabolism to promote colon tumorigenesis via an IL-17-STEAP4-XIAP axis. Nat Commun. 2020;11(1):900. Published 2020 Feb 14. doi:10.1038/s41467-020-14698-y

rabbit anti-GAPDH (1:2000, Cell Signaling 5174), WB: Kang L, Yu H, Yang X, et al. Neutrophil extracellular traps released by neutrophils impair revascularization and vascular remodeling after stroke. Nat Commun. 2020;11(1):2488. Published 2020 May 19. doi:10.1038/s41467-020-16191-y

rabbit anti-beta-Actin-HRP (1:2000, Cell Signaling 5125), WB: Kong LR, Ong RW, Tan TZ, et al. Targeting codon 158 p53-mutant cancers via the induction of p53 acetylation. Nat Commun. 2020;11(1):2086. Published 2020 Apr 29. doi:10.1038/s41467-020-15608-y

Rabbit anti-Claudin5 (1:400, Abcam 53765), IF: Rajani RM, Quick S, Ruigrok SR, et al. Reversal of endothelial dysfunction reduces white matter vulnerability in cerebral small vessel disease in rats. Sci Transl Med. 2018;10(448):eaam9507. doi:10.1126/scitranslmed.aam9507

Rabbit anti-Hey1 (1:500, Abcam 22614), WB: Kung-Chun Chiu D, Pui-Wah Tse A, Law CT, et al. Hypoxia regulates the mitochondrial activity of hepatocellular carcinoma cells through HIF/HEY1/PINK1 pathway. Cell Death Dis. 2019;10(12):934. Published 2019 Dec 9. doi:10.1038/s41419-019-2155-3

Mouse anti-CD31 (1:50, Dako 0823), IF: Pekkonen P, Alve S, Balistreri G, et al. Lymphatic endothelium stimulates melanoma metastasis and invasion via MMP14-dependent Notch3 and β1-integrin activation. Elife. 2018;7:e32490. Published 2018 May 1. doi:10.7554/eLife.32490

Mouse anti-alpha-SMA (1:300, Dako M0851), WB: Demircioglu F, Wang J, Candido J, et al. Cancer associated fibroblast FAK

regulates malignant cell metabolism. Nat Commun. 2020;11(1):1290. Published 2020 Mar 10. doi:10.1038/s41467-020-15104-3

Rabbit anti-NG2 (1:100, Millipore 5384): IF: Sobrino A, Phan DT, Datta R, et al. 3D microtumors in vitro supported by perfused vascular networks. Sci Rep. 2016;6:31589. Published 2016 Aug 23. doi:10.1038/srep31589

Mouse anti-GFAP (1:100, Millipore 360), IF: Kim DY, Park JA, Kim Y, et al. SALM4 regulates angiogenic functions in endothelial cells through VEGFR2 phosphorylation at Tyr1175. FASEB J. 2019;33(9):9842-9857. doi:10.1096/fj.201802516RR

Rabbit anti-VE-cadherin (1:400, Enzo ALX-210-232), IF: Antfolk D, Sjöqvist M, Cheng F, et al. Selective regulation of Notch ligands during angiogenesis is mediated by vimentin. Proc Natl Acad Sci U S A. 2017;114(23):E4574-E4581. doi:10.1073/pnas.1703057114

Goat anti-Dll4 (1:50, R&D AF1389), IF: Fu R, Lv WC, Xu Y, et al. Endothelial ZEB1 promotes angiogenesis-dependent bone formation and reverses osteoporosis. Nat Commun. 2020;11(1):460. Published 2020 Jan 23. doi:10.1038/s41467-019-14076-3

rat anti-mouse-CD31(1:200, Dianova, DIA-310), IF: Esteban S, Clemente C, Koziol A, et al. Endothelial MT1-MMP targeting limits intussusceptive angiogenesis and colitis via TSP1/nitric oxide axis. EMBO Mol Med. 2020;12(2):e10862. doi:10.15252/emmm.201910862

mouse anti-CCND1 (1:1000, Santa Cruz Biotech, sc-8396), WB: Perra A, Kowalik MA, Cabras L, et al. Potential role of two novel agonists of thyroid hormone receptor-β on liver regeneration. Cell Prolif. 2020;53(5):e12808. doi:10.1111/cpr.12808

rabbit anti-Slug (1:50, Santa Cruz Biotech 15391). Yin X, Zhang BH, Zheng SS, et al. Coexpression of gene Oct4 and Nanog initiates stem cell characteristics in hepatocellular carcinoma and promotes epithelial-mesenchymal transition through activation of Stat3/Snail signaling. J Hematol Oncol. 2015;8:23. Published 2015 Mar 11. doi:10.1186/s13045-015-0119-3

Eukaryotic cell lines

Policy information about cell lines

Cell line source(s)	CMT-93 (ATCC), MC-38 (NCI), B16 (ATCC), HUVEC were generated in lab.
Authentication	All tumor lines were purchased recently for the purposes of the study and were verified by the manufacturer. All of our lines are also regularly validated by STR analysis at the University of Arizona Genetics Core (https://uagc.arl.arizona.edu/node/27))
Mycoplasma contamination	Cells were regularly tested for Mycoplasma and the ones used in this study were negative for mycoplasma contamination
Commonly misidentified lines (See ICLAC register)	None.

Animals and other organisms

Policy information about studies involving animals; ARRIVE guidelines recommended for reporting animal research

Laboratory animals	B6;129S1-Snai2tm2Grid/J (Slug knockout, SlugKO) mice, B6.FVB-Tg(Cdh5-cre)7Mlia/J mouse (VEcad-Cre), Dll4tm1Jrt/ICR, C57BL6/J, ICR. For all studies, both genders were included. For tumor and related experiments, mice 20 to 24 weeks of age were used. For retina experiments, mice 2 to 15 days of age were used. For Miles assay, mice 6-12 weeks of age were used.
Wild animals	This study did not involve wild animals.
Field-collected samples	This study did not invovle samples collected from the field
Ethics oversight	All animal studies were approved by and conducted in compliance with University of California, Irvine IACUC regulations.

Note that full information on the approval of the study protocol must also be provided in the manuscript.

Flow Cytometry

Plots

Confirm that:

- [x] The axis labels state the marker and fluorochrome used (e.g. CD4-FITC).
- [x] The axis scales are clearly visible. Include numbers along axes only for bottom left plot of group (a 'group' is an analysis of identical markers).
- [x] All plots are contour plots with outliers or pseudocolor plots.
- [x] A numerical value for number of cells or percentage (with statistics) is provided.

Methodology

Sample preparation	Neonatal mice aged between P8 to P12 were used in this experiment. Fresh retinas from 2-4 pups of WT, Slug heterozygous or SlugKO mice were dissected in cold PBS and pooled. After dissection, retinas were placed into 1mL of digestion buffer containing 1mg/mL collagenase type II in 20% LG-DMEM and incubated at 37°C for 20 minutes. After dissection, the cells were passed through a 70µm cell strainer, spun down and resuspended in staining buffer containing 1% BSA.
Instrument	FACS ARIA II
Software	No additional software were used for cell collection and analysis
Cell population abundance	CD31+ cells were around 1% of live singlets. Cells were back-gated to check for purity.
Gating strategy	Dead cells and cellular debris were excluded by FSC-A and SSC-A. Doublets were excluded by FSC-A and FSC-H. Non-stained cells were used as negative control and to gate CD31 negative cells. CD31 stained samples were used to gate CD31 positive cells. First 10,000 cells were used to establish the gates before sorting.

☒ Tick this box to confirm that a figure exemplifying the gating strategy is provided in the Supplementary Information.
